# Association of killer cell immunoglobulin-like receptors with endemic Burkitt lymphoma in Kenyan children

**DOI:** 10.1038/s41598-021-90596-7

**Published:** 2021-05-31

**Authors:** Beatrice M. Muriuki, Catherine S. Forconi, Peter O. Oluoch, Jeffrey A. Bailey, Anita Ghansah, Ann M. Moormann, John M. Ong’echa

**Affiliations:** 1 West African Center for Cell Biology of Infectious Pathogens, College of Basic and Applied Sciences, University of Ghana, Accra, Ghana; 2 Center for Global Health Research, Kenya Medical Research Institute, Kisumu, Kenya; 3 Division of Infectious Diseases and Immunology, Department of Medicine, University of Massachusetts Medical School, Worcester, MA USA; 4 Department of Pathology and Laboratory Medicine, Warren Alpert Medical School, Brown University, Providence, RI USA; 5 Noguchi Memorial Institute for Medical Research, College of Health Sciences, University of Ghana, Legon, Accra, Ghana

**Keywords:** Cancer, Immunology, Molecular biology

## Abstract

Endemic Burkitt lymphoma (eBL) is an aggressive pediatric B cell lymphoma, common in Equatorial Africa. Co-infections with Epstein-Barr virus (EBV) and *Plasmodium falciparum,* coupled with *c-myc* translocation are involved in eBL etiology. Infection-induced immune evasion mechanisms to avoid T cell cytotoxicity may increase the role of Natural killer (NK) cells in anti-tumor immunosurveillance. Killer immunoglobulin-like receptor (KIR) genes on NK cells exhibit genotypic and allelic variations and are associated with susceptibility to diseases and malignancies. However, their role in eBL pathogenesis remains undefined. This retrospective study genotyped sixteen KIR genes and compared their frequencies in eBL patients (n = 104) and healthy geographically-matched children (n = 104) using sequence-specific primers polymerase chain reaction (SSP-PCR) technique. The relationship between KIR polymorphisms with EBV loads and eBL pathogenesis was investigated. Possession of ≥ 4 activating KIRs predisposed individuals to eBL (OR = 3.340; 95% CI 1.530–7.825; *p* = 0.004). High EBV levels were observed in Bx haplogroup (*p* = 0.016) and AB genotypes (*p* = 0.042) relative to AA haplogroup and AA genotype respectively, in eBL patients but not in healthy controls. Our results suggest that KIR-mediated NK cell stimulation could mute EBV control, contributing to eBL pathogenesis.

## Introduction

Endemic Burkitt lymphoma (eBL) is the quintessential Epstein-Barr Virus (EBV)-associated B cell malignancy in pediatric patients within Africa and Papua New Guinea^1^. In Africa, eBL has the highest incidence in areas where *Plasmodium falciparum *(*Pf*) malaria is common, hence repeated interaction of *Pf-*infected red blood cells with EBV-infected B cells is postulated to result in eBL oncogenesis^1, 2^. The proposed mechanism of eBL development involves a combination of activation-induced cytidine deaminase (AID)-associated *c-myc* chromosomal translocation, modulation of host T cell immunity to EBV antigens, monoclonal expansion of B cells infected with EBV, and reactivation of EBV, resulting in increased viremia^3^. Cells infected with EBV down-regulate the expression of human leukocyte antigen (HLA) to evade recognition by HLA-restricted cytotoxic CD8 + T cells^4^. However, this immune evasion mechanism, i.e. ‘missing-self’ should render them susceptible to killing by natural killer (NK) cells^4^. NK cells constitute the body's first line of defense against viral infections and tumor cells^5^. They are identified by the expression of CD56, a neural cell adhesion molecule 1 (NCAM-1) belonging to the immunoglobulin supergene family^6^. This molecule mediates cell to cell interactions and its surface expression levels vary with cell maturation^6^. Consequently, there are two major NK cell populations: CD56^bright^ are mainly cytokine-producing^7^, while CD56^dim^ acquire additional CD16 and killer immunoglobulin-like receptors (KIRs) receptors, which enhance their cytolytic activities^7, 8^. Other studies have reported accumulation of CD56 ^negative^ CD16 ^positive^ NK cell subsets in eBL patients^9^, and in HIV-infected individuals^10^. NK cell anti-viral and anti-tumor activities are partly regulated by inhibitory (iKIRs) and activating (aKIRs) KIRs, which are also expressed by some TCR-γδ, CD8^+^, and CD4^+^ T cells^11–13^. KIRs interact with various HLA class I ligands on target cells^14^. Tumor cells lacking ligands for inhibitory and activating KIRs do not stimulate NK cells response^15^. Additionally, NK cells do not kill healthy cells when only the inhibitory receptors are ligated to HLA-I ligands on the target cells, since there is no activating signal generated^15^. Down-regulation of HLA-I in target cells by viral infections or neoplastic transformation results in a lack of ligation of inhibitory NK cell receptors to their ligands, hence the absence of NK cell inhibition. Instead, only the activating NK cell receptors are ligated to activating ligands, resulting in NK cell stimulation to kill target cells^16^. The outcome of the interaction of NK cells with tumor cells containing ligands for both inhibitory and activating receptors depends on the balance of the strength of signals generated^15^.

**Figure 1:**
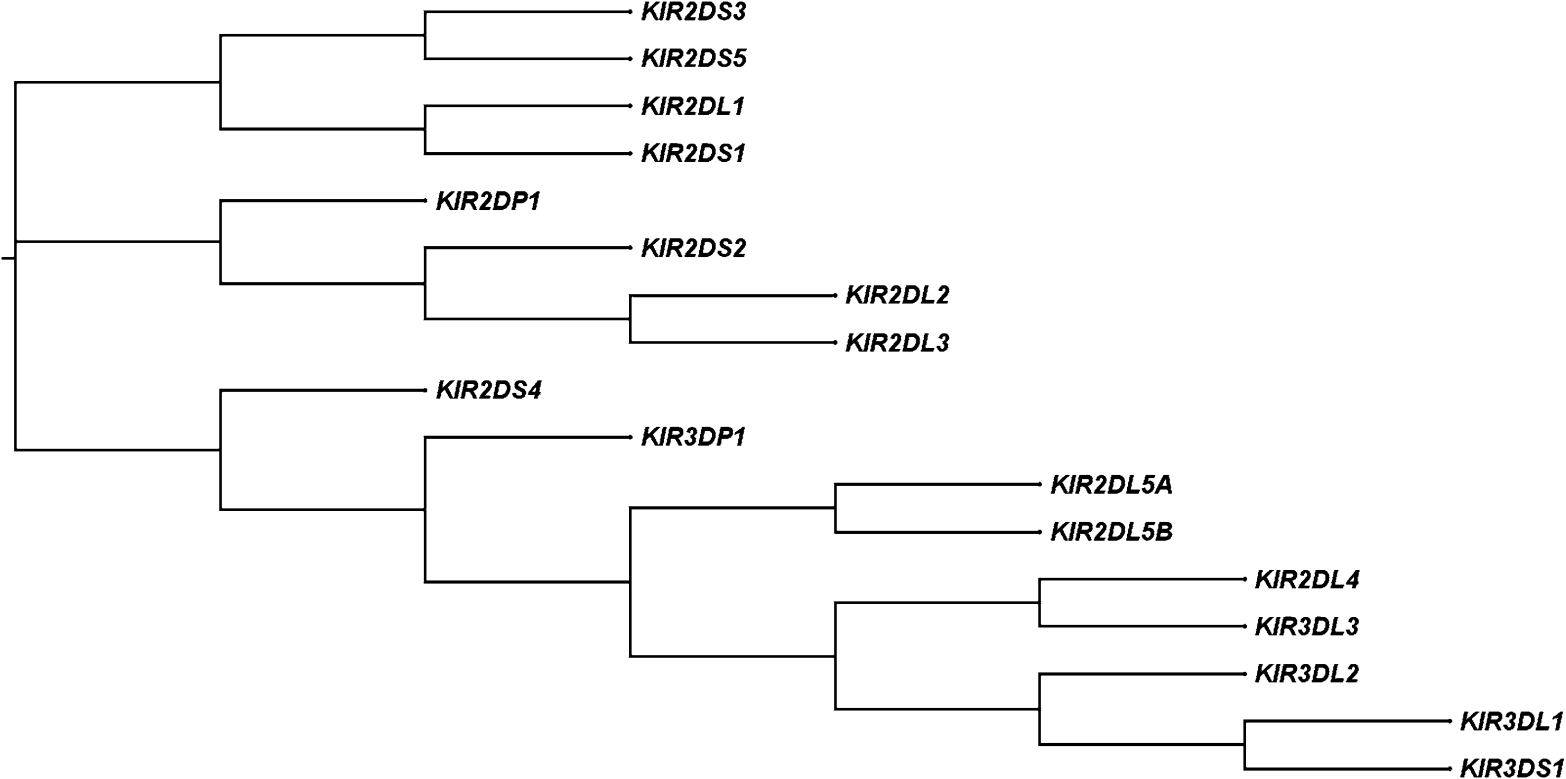
Phylogenetic relationship of human killer immunoglobulin-like receptor gene showing three clades. The Neighbor-Joining (NJ) tree was generated using publicly available KIR DNA sequences in Clustal Omega (EMBL-EBI) with default settings.

The KIR gene family comprises rapidly evolving genes present in all primates^17^. The genes contain two (2D) or three (3D) domains in the extracellular region, with a short (S) or a long (L) cytoplasmic tail^18^. The KIR genes *2DL1, 2DL2/2DL3, 2DL5, 3DL1, 3DL2*, and *3DL3* have a long tail with an inhibitory motif. Short cytoplasmic tailed KIRs have activating motifs and include *2DS1, 2DS2, 2DS4, 2DS3/2DS5*, and *3DS1*^18^. KIR2DL4 is the only long-tailed receptor with both inhibitory and activating motifs^19^. There are two pseudogenes, *2DP1* and *3DP1*^20^ (Fig. 1). These genes are arranged in a head-to-tail order in the long arm of chromosome 19 (19q13.4), within the Leukocyte Receptor Complex (LRC)^21^. Each KIR gene is 10–16 kb in length, with a 2 kb sequence separating each gene pair, except a 14 kb sequence that occurs upstream of *KIR2DL4*^21^. The expression of KIR genes varies between NK cell subsets and is controlled by four types of promoters^22^. CD56^dim^ NK cells express all KIR genes except *3DL3*^22^. *2DL4* occurs on both CD56^bright^ and ^-dim^ NK cells in a non-variegated manner^22^. Some KIR genes demonstrate variations in their sequences, for example, *KIR2DS4* has a 22 base pair (bp) deletion in the second extracellular domain which results in a non-functional gene^21^. A deletion of one base-pair in exon 4 of *KIR2DP1* introduces a stop codon, resulting in a pseudogene^23^. Another pseudogene, *KIR3DP1* has a deletion of 1.5 kb that removes exon 2. There are no transcripts for the 2 pseudogenes^23^. *KIR2DL5* has two variants A and B, encoded by different loci^24^. *2DL5B* is in the centromeric region, while *2DL5A* occurs in the telomeric region^25^.

Polymorphisms within the KIR locus result from gene content, allelic, and copy number variations^26^. Based on gene content and copy number, KIRs are grouped into inhibitory haplotype A and activating haplotype B^27^. The haplotypes are further subdivided into AA and Bx genotypes, where x can be either A or B^28^. There are more than 500 different Bx groups in the database (http://www.allelefrequencies.net)^28^. KIR genotype AA is homozygous for the haplotype A and contains *3DL3, 2DL3, 2DP1, 2DL1, 3DP1, 2DL4, 3DL1, 2DS4*, and *3DL2* genes^21^. Activator haplotype B has a variable number of activating KIRs, and comprise of *2DS2, 2DL2, 2DL5B, 2DL1, 2DP1, 3DP1, 3DL3, 2DL4, 3DS1, 2DL5A, 2DS3/2DS5, 2DS1, 2DS4, 2DS3/2DS5* and *3DL2* genes^21^. This haplotype has a Bx group containing one (AB heterozygous) or two (BB homozygous) genotypes. Genotype BB does not have one or more of the group A KIR genes. All the remaining genotypes in haplotype B are defined as AB^29^. *3DL3, 3DL2, 2DL4*, and *3DP1* are framework genes, hence they appear in all haplotypes^23^. KIR haplotypes are split into centromeric A or B (cA, cB) and telomeric A or B (tA, tB) halves^30, 31^. Both cA, cB and tA, tB regions exhibit an even balance in East Africa population^32^. Classification of KIR based on presence/absence of a gene generates eight telomeric regions (tA01, tB01, tB02, tB03, tB04, tB05, tB06 and tB07) and nine centromeric regions (cA01, cA02, cA03, cB01, cB02, cB03, cB04, cB05 and cB06)^33^. *KIR2DL5, 2DS5*, and *2DS3* are duplicated and can occur in centromeric and/or telomeric locations^34^. Genes occurring in different regions of the KIR complex may undergo homologous recombination, resulting in expanded and contracted haplotypes^34, 35^ The B content score is the sum of cenB and/or telB motifs in each genotype^36^. The Bx group can be classified further into four subsets, by considering two gene clusters; T4, containing *KIR2DL5-3DS1-2DS1-2DS5* genes, and C4, which has *KIR2DL2-2DS2-2DS3-2DL5* genes. The C4T4 contains both C4 and T4 genes, while the C4Tx subset has C4 but lacks T4 genes. CxT4 lacks C4 genes, thus it contains T4 genes. The absence of both C4 and T4 genes results in CxTx subset^29, 37^.

Studies have suggested that specific KIRs influence the generation of either inhibitory versus activating signals. A balance between these signals determines whether NK cells bypass or kill viral-infected or tumor cells^38^. Consequently, these signals can influence an individual's susceptibility to diseases and malignancies^16, 30, 39, 40^. The presence of certain KIRs has been associated with cancer pathogenesis. For instance, an increased number of activating KIRs predispose individuals to EBV-related nasopharyngeal carcinoma (NPC)^16^, whereas the presence of genotype B, which mainly contains activating KIRs is associated with gastric cancer lesions^30^. In contrast, the Bx haplogroup protects against colorectal adenocarcinoma^41^. However, there is little understanding of the impact of KIR polymorphisms on eBL pathogenesis. Therefore, to improve our understanding of how KIR genes may contribute to eBL pathology, we performed KIR genotyping using commercially available kits and analyzed the haplotype, genotypes, centromere-telomere regions, Bx subsets, and B score contents in eBL patients and healthy controls. Given the strong link between EBV and eBL^42^, we further evaluated the association of haplogroups AA/Bx and genotypes AA, AB, and BB with EBV loads, to determine viral control.

## Results

### KIR genes

To characterize the frequencies of KIR genes in the study population, we genotyped the genes responsible for inhibitory signals (*2DL1, 2DL2/2DL3, 2DL5, 3DL1*), activating signals (*2DS1, 2DS2, 2DS4, 2DS3/2DS5*, and *3DS1*), the framework and pseudogenes (2DL4, *3DL2*, *3DL3, 2DP1* and *3DP1*) from genomic DNA using sequence-specific primers polymerase chain reaction (SSP-PCR) technique. KIR genotypes were classified based on the presence or lack of each gene locus and were analyzed to determine differences in their frequencies between eBL patients and healthy controls (HC). The genes *KIR3DP1, KIR2DP1*, *KIR2DL1, KIR2DL4, KIR3DL2* and *KIR3DL3* occurred at a frequency ≥ 99% and were excluded from the association analysis. The KIR genes were not statistically different between the study groups (Table 1).

**Table 1:** Analysis of the association of KIR genes with endemic Burkitt lymphoma by multivariate logistic regression. Comparisons were made with healthy control as the reference group. *p* ≤ 0.05 is considered statistically significant. *p* ≤ 0.05 and the OR (95% CI) were adjusted by age and sex.

Genes	eBL n = 104 (%)	HC n = 104 (%)	Odds Ratio (95%Confidence Interval)	*p*-value
**Inhibitory genes**				
*2DL2*	64 (61.5)	54 (51.9)	1.246 (0.641–2.427)	0.516
*2DL3*	90 (86.5)	91 (87.5)	1.267 (0.447–3.737)	0.658
*2DL5All*	70 (67.3)	60 (57.7)	1.283 (0.654–2.532)	0.468
*2DL5A*	16 (15.4)	11 (10.6)	1.762 (0.699–4.540)	0.232
*2DL5B*	61 (58.7)	55 (52.9)	1.118 (0.580–2.154)	0.738
*3DL1*	102 (98.1)	102 (98.1)	0.303 (0.013–3.412)	0.345
**Activating genes**				
*2DS1*	27 (26.0)	18 (17.3)	1.383 (0.633–3.040)	0.416
*2DS2*	54 (51.9)	47 (45.2)	1.136 (0.589–2.190)	0.702
*2DS3*	20 (19.2)	21 (20.2)	0.840 (0.341–2.023)	0.699
*3DS1*	16 (15.4)	17 (16.3)	1.124 (0.472–2.657)	0.790
*2DS5*	58 (55.8)	44 (42.3)	1.394 (0.718–2.704)	0.324
*2DS4ins*	68 (65.4)	70 (67.3)	0.887 (0.444–1.773)	0.733
*2DS4del*	67 (64.4)	67 (64.4)	1.364 (0.689–2.748)	0.378

### KIR haplotypes and genotypes

**Figure 2:**
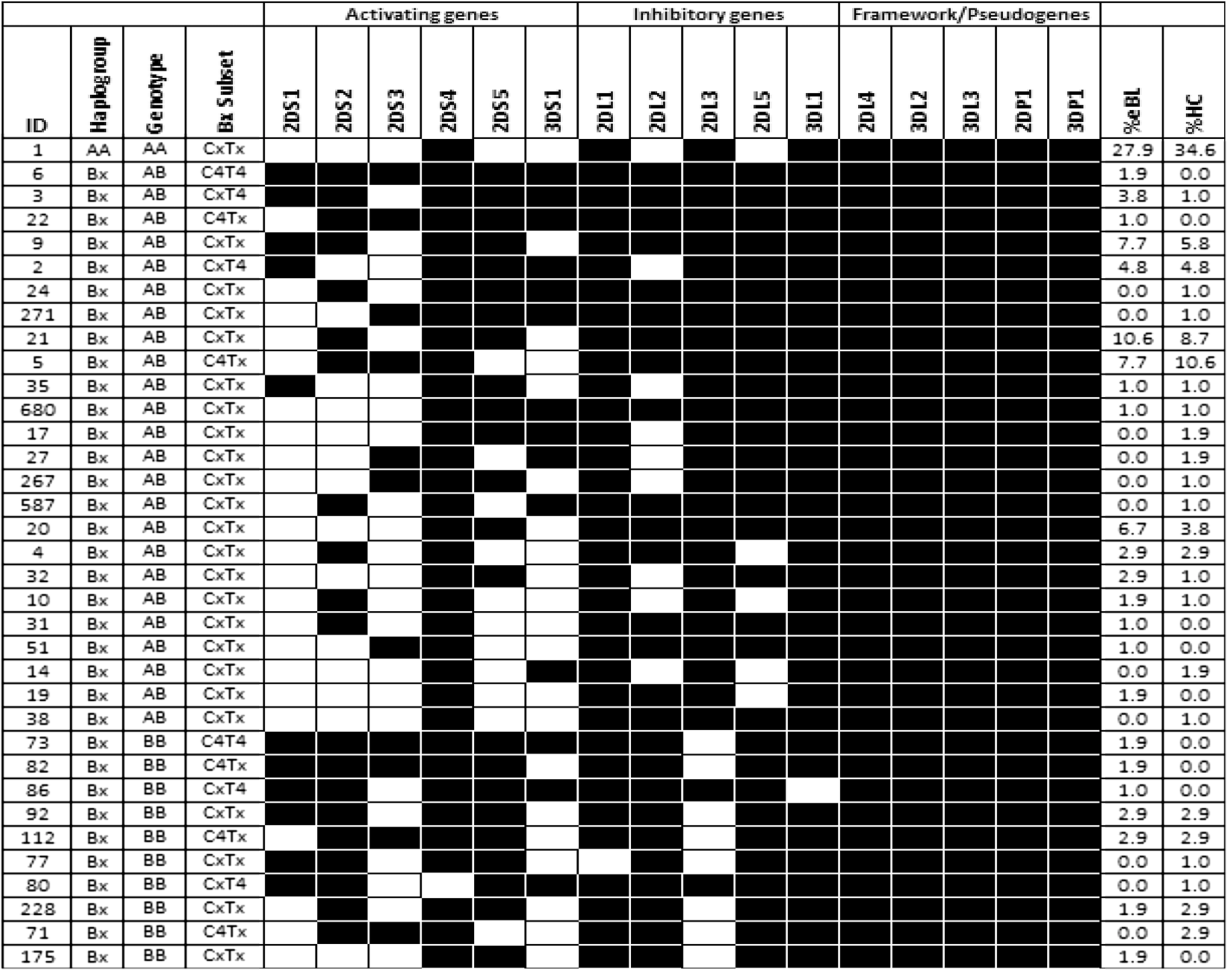
The occurrence of KIR genotypes in the study population. Thirty-five different KIR genotypes were observed in the 208 persons. The genotypes differed from each other by the presence of (black box) or absence (open box) of KIR genes. The variants *KIR2DL5A* and *2DL5B* were considered as *KIR2DL5* while *KIR2DS4* mutant and *2DS4* full length were considered as *KIR2DS4*, we analyzed a total of 16 KIR genes. The data are expressed as percentage frequency, obtained by dividing the number of individuals having the genotype by the number of individuals in the studied group. Genotype ID reference numbers were acquired from the KIR genotype database (http://www.allelefrequencies.net).

In the studied population, the haplotypes A and B occurred at frequencies of 56.7% vs. 60.6% and 43.3% vs 39.4% in eBL patients and HC respectively. The haplotypes were grouped into haplogroup AA (27.9% vs. 34.6%) and Bx (72.1% vs. 65.4%) for eBL patients and HC respectively (Table 2). There were 35 different haplogroups in the study population, based on the allele frequencies database (http://www.allelefrequencies.net)^28^ (Fig. 2). Out of these, 15 were identified in both cases and controls, fourteen had frequencies > 1.0%; representing 88.5% of the population, while eighteen had frequencies > 1.0% representing 94.2% of the healthy controls and eBL patients respectively. The remaining haplogroups (17 in eBL and 21 in HC) were rare, with frequencies ≤ 1.0%. The haplogroups were subdivided further into genotypes AA, AB, or BB according to the gene content. Among 104 eBL patients, 29 were genotypes AA (27.9%), 60 were AB (57.7%) while 15 were BB (14.4%). All the AA genotypes had ID 1. Among 104 HC, 36 were genotypes AA (34.6%), 54 were AB (51.9%) while 14 were BB (13.5%). The distribution of the KIR genotypes among the study groups was not statistically significant (Table 2).

**Table 2:** Comparison of haplotypes, genotypes, linkage groups, and the number of activating KIRs between endemic Burkitt lymphoma patients and healthy controls.

Gene		eBL n = 104	HC n = 104	OR (95% CI)			*p*-value
		N (%)	N (%)				
**Haplotype**							
A		118 (56.7)	126 (60.6)	0.853 (0.487–1.487)			0.673
B		90 (43.3)	82 (39.4)				
**KIR haplogroup and genotype frequencies**							
AA		29 (27.9)	36 (34.6)	0.730 (0.400–1.308)			0.370
Bx		75 (72.1)	68 (65.4)				
AB		60 (57.7)	54 (51.9)	1.263 (0.733–2.190)			0.486
BB		15 (14.4)	14 (13.5)	1.083 (0.511–2.297)			1.000
**KIR Bx subgroup (Linkage group) frequencies**							
C4T4		4 (3.8)	0	NA			NA
CxT4		10 (9.6)	7 (6.7)	1.474 (0.554–3.818)			0.614
C4Tx		14 (13.5)	17 (16.3)	0.796 (0.386–1.716)			0.698
CxTx		76 (73.1)	80 (76.9)	0.814 (0.431–1.510)			0.631
C4 gene-cluster		18 (17.3)	17 (16.3)	1.071 (0.533–2.178)			1.000
T4 gene-cluster		14 (13.5)	7 (6.7)	2.156 (0.807–5.495)			0.166
**Number of activating KIRs**							
≥ 4		25 (24.0)	9 (8.7)	3.340 (1.530–7.825)			**0.004**
< 4		79 (76.0)	95 (91.3)				

*p* ≤ 0.05 are considered statistically significant; based on the two-tailed Fisher's exact test. The haplotype A and B were obtained as follows; haplotype A = 2NAA + NAB/2n and haplotype B = 2NBB + NAB/2n. The NAA, NAB, and NBB are the numbers of AA, AB, and BB genotypes, n = total number of individuals^37, 43^.

### KIR centromeric and telomeric distribution

The KIR gene contents vary in the centromeric and telomeric regions. To investigate these differences in our study population, genotypes AA and Bx were grouped into centromeric and telomeric contents^31^. A total of 6 centromeric and 2 telomeric genetic regions were reported. There were no significant differences in these regions when comparing eBL patients with the control group (Supplementary Table S1).

### KIR B score

To evaluate the involvement of B motifs in eBL development, the AA and Bx genotypes were investigated according to the distribution of B content in the centromeric (cB) and telomeric (tB) regions. A score of zero was more common in healthy controls while a score of one was frequent in eBL patients (44.2% vs. 38.5 and 43.3 vs. 50.0% respectively) (Supplementary Table S2). However, the observed differences were not statistically significant.

### Bx Subsets and the number of activating KIRs

We observed that all the four Bx subsets (C4T4, CxT4, C4Tx, CxTx) were present in the study population (Table 2). C4T4 had the least frequency, with no representation among the healthy individuals. CxTx had the highest frequency in both eBL patients and healthy controls. Further, in order to investigate possible differences in the number of KIRs, we compared the iKIRs and aKIRs in cases and controls. There were an increased proportion of eBL patients with ≥ 4 aKIRs relative to HC.

### KIR haplotypes, genotypes, and EBV Viral loads

**Figure 3:**
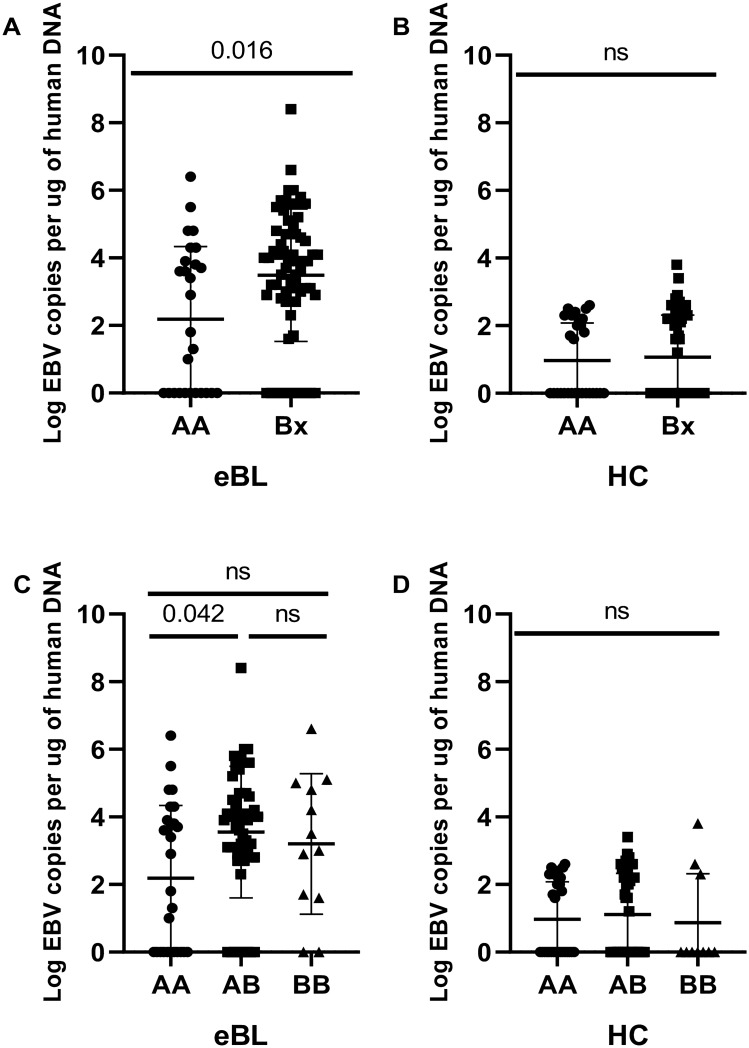
EBV load stratified by AA/Bx haplogroups and AA, AB, and BB genotypes. The EBV levels were compared for eBL patients (n = 93), and healthy controls (n = 80) after stratification by AA/Bx haplogroups (**A**,**B**) and AA, AB, and BB genotypes (**C**,**D**). Significant differences in EBV viral loads were associated with AA/Bx haplogroups and AA/AB genotypes in eBL patients but not in healthy controls; based on Mann–Whitney test and one way Kruskal–Wallis statistic respectively. The *p*-value in C was statistically significant; hence pair-wise comparisons were assessed by Dunn's test. (*p < 0.05). ns = not significant.

We compared EBV viral loads and observed significantly higher viremia in children with eBL (median 6496.571 EBV copies/μg of DNA) compared to healthy children from the malaria holoendemic region (median 202.697 EBV copies/μg of DNA) (*p*-value <0.0001). Next, we investigated whether the EBV loads differed between the haplotypes and genotypes. Considering the KIR haplotypes and genotypes, we observed significant differences in EBV load in eBL patients but not in healthy controls (Fig. 3).

## Discussion

KIR gene polymorphisms predispose individuals to various malignancies associated with viruses^16^. However, few studies have evaluated the role of such polymorphisms in eBL etiology. To address this issue, we evaluated the association of KIR genes with eBL development. The most common KIR genotype in our study population was homozygous A, with the genotype id AA1. This genotype has previously been shown to be the most frequent in all worldwide populations, including Africans^44–46^. Interestingly, while its frequency in healthy controls was consistent with the expected frequency in African populations (35.6%)^45^, the representation was lower in eBL patients (27.9%). However, such variations have been reported in a few African populations, from 12.0% in the Xhosa population of South Africa, 28.1% in the Ugandan population, and 42.0% in Senegal^44, 47, 48^. In our study, the Bx genotype was highly variable, with a frequency range of 0.0–10.6%. This genotype consists of two haplotypes; AB and BB. Most of the study participants were heterozygous AB, and there was a very low frequency of homozygous BB. The considerable diversity for Bx but not AA genotypes may be a result of copy number variation^33^ due to selection pressure from environmental, climatic, chronic, and infectious diseases that have prevailed in our study population for many years^40^. Recently, malaria has been shown to drive selection for this haplotype in a Ugandan population^49^. In our study, there were more eBL patients carrying ≥ 4 aKIRs compared to healthy controls; suggesting that individuals with ≥ 4 aKIRs may have a high risk of developing eBL. Consistent with our findings, an increased number of aKIRs predispose individuals to colorectal adenocarcinoma, human papillomavirus-associated cervical cancer and EBV-associated nasopharyngeal carcinoma^16, 39, 50^. The role of the number of aKIRs in the etiology of cancers is explained by two hypotheses^39^. First, an increased number may protect individuals against cancers, due to enhanced cytolysis of tumor cells, resulting from increased NK cell activation^30^. In contrast, increased immune activation of NK cells by aKIRs may cause non-specific inflammatory responses, such as oxidative DNA damage^16, 30^. Such responses may increase the risk of cancer development^30^. Therefore, considering the second hypothesis, our findings raise a possibility that an increasing number of aKIRs coupled with repeated infections with *Plasmodium falciparum* in our study population^51^ could be associated with increased NK cell activation resulting in inflammation-associated oncogenesis.

The study participants had a higher frequency of centromeric B region and a lower frequency of telomeric B region. Similar observations were reported in a Ugandan population^44^. Generally, cenB region is common in the African population relative to telB region^44^. The number of B motifs in the centromere and telomere regions influences NK cell activation^30^. Subsequently, the B motif is associated with disease outcome^30^. In this study, we evaluated how the number of KIR B gene motifs of centromeric or telomeric origin influences eBL development. There were no significant differences in the B score when comparing eBL patients with the control group.

Previous studies have reported that children living in malaria holoendemic areas experience primary EBV infection at an early age compared to children residing in areas with lower incidences of malaria^52^. In addition, repeated exposure to malaria is associated with poor EBV control, hence higher viremia^53^. Consistent with these findings, we observed that eBL patients had higher median EBV loads relative to healthy controls (6496.571 versus 202.697 EBV copies/μg of DNA), respectively, *p-*value < 0.0001). The EBV levels were significantly different when considering the KIR haplogroup and genotypes in children with eBL but not in healthy controls, with higher EBV loads observed in Bx relative to AA haplogroup. Considering the genotypes, EBV load differed between AA and AB. NK cells are essential in the control of infections associated with viruses^54^. Their subsets expand upon infection with herpes viruses^55^, and the proliferation positively correlates with EBV viral loads^55^. Individuals deficient in NK cells are predisposed to herpes viruses-associated infections^56^. The persistence of viruses in an individual may cause chronic recruitment and activation of NK cells, up to when in some individuals; the NK cell activation is deregulated. Therefore, increased viral load in Bx relative to AA haplogroup in eBL could be related to continuous stimulation and subsequent loss of NK cell control of EBV. A previous study reported the accumulation of dysfunctional CD56 negative CD16 positive subset of NK cells in eBL patients^9^. Consistent with these findings, our results raise the possibility that activation of NK cells that are mediated by KIRs may impair NK cell functions in our study population^9^. Further studies are required to confirm this observation and the role of KIR-expressing T cells^11–13^.

A limitation of our study was the small sample size and convenience sampling of healthy controls which led to them being younger than eBL cases. However, since KIR genotypes do not change with age, we don’t believe this biased our findings. In addition, our conclusion is restricted by a lack of HLA ligands data, as KIR/HLA combinations influence NK cell activity. As KIRs can vary at the gene or allele level, we only investigated the presence or absence of each KIR gene, and we could not evaluate allelic and copy number variations that can impact NK cell functions. A previous study reported decreased expression of *KIR2DL1/S1* inhibitory/activation marker and increased expression of *KIR3DL1* in children exposed to malaria and eBL patients relative to healthy children^9^, hence future studies will need to assess the mechanistic implications of KIR proteins and gene expression profiles in eBL pathogenesis. We acknowledge that whereas our findings suggest a possible association of the Bx haplogroup and increased number of aKIRs with EBV load and eBL pathogenesis respectively, there is a need for a larger validation cohort and functional studies to confirm their biological relevance.

## Materials and methods

### Study site and subjects

The study enrolled 208 children; 104 eBL patients, and 104 healthy individuals. Patients with eBL were children aged 0–14 years old, who were enrolled at Jaramogi Oginga Odinga Teaching and Referral Hospital (JOOTRH), located in Kisumu County, western Kenya. JOOTRH is one of the two regional referral centers for childhood cancer cases in western Kenya. Morphologic diagnosis of eBL was performed by staining fine-needle aspirates (FNA) with Giemsa/May-Grünwald and observed under a microscope^57^. The control group consisted of convenience samples selected from healthy non-eBL children, aged 0–12 years old, living in the same malaria-holoendemic regions of western Kenya as the eBL patients. Convenience sampling did not affect our results since KIR genotypes do not differ by age.

### DNA extraction and KIR genotyping

Genomic DNA was isolated from 200 μl of blood using Qiagen QIAamp DNA Mini Kit (Valencia, CA, USA), following the manufacturer’s instructions, and frozen at − 20 °C until genotyping. The samples were analyzed for KIR gene content^58^. A commercially available KIR genotyping sequence-specific Primers (SSP) kit (Miltenyi, Biotec, Inc, Germany), was used to test for the presence or absence of KIRs generating inhibitory signals (*KIR2DL1, 2DL2, 2DL3, 2DL5A, 2DL5B, 2DL5* (*A* and *B*) *3DL1*), activating KIRs (*2DS1, 2DS2, 2DS3, 2DS4del, 2DS4ins, 2DS5*, and *3DS1*) and the framework and pseudogenes (*2DL4, 3DL2, 3DL3*, *2DP1* and *3DP1*) following the manufacturer’s recommendations. The amplified sequences were examined by electrophoresis in 2% agarose gel stained with SYBR Safe (Invitrogen, Burlington, ON, Canada) and visualized on a UV transilluminator using a gel documentation system (ChemiDoc, BioRad) for the presence or lack of amplicons specific to each gene, according to the manufacturer’s instructions (See full-length gel in Supplementary Fig. 1 online).

### Definitions for KIR gene content polymorphisms

KIR polymorphisms were analyzed by determining the presence or absence of 16 KIRs genes; 2 pseudogenes, 8 inhibitory, and 6 activating KIR genes. The KIR haplotypes were classified into AA (inhibitory) and Bx (activator), where x can be A or B. The homozygote AA genotype was defined by the absence of *KIR2DL2, 2DL5* (*2DL5A* and *B*, *2DL5A*, *2DL5B*) *2DS1, 2DS2, 2DS3, 2DS5*, and *3DS1* genes. Individuals in the Bx genotype contained at least one of the genes above^28^. The genes *2DL4, 3DP1, 3DL2*, and *3DL3* are framework genes^23^. The genotypes AA and Bx were evaluated according to the distribution of the centromeric and telomeric genes and the B content score as previously reported^31^.

### EBV Load

Epstein-Barr virus load was determined by quantitative Polymerase Chain Reaction (qPCR)^59^. Briefly, amplification of genomic DNA was done in a Bio-Rad CFX96 Real-Time System with C1000 Thermo Cycler base for the primers and probes (BioRad Laboratories, Hercules, CA), following the manufacturer’s instructions. The PCR amplification conditions were: 180 s at 95 °C, 10 s at 95 °C, 30 s at 63.5 °C- plate read, 10 s at 95 °C (39 times).

### Statistical analysis

The frequencies of KIR genes, haplotypes, genotypes, B score, and centromere-telomere gene content in eBL patients were compared with the healthy controls. Differences between KIR genes and EBV loads were assessed by Fisher’s exact test using Graphpad Prism version 8.0.2 software (GraphPad Software, La Jolla, CA). Comparisons of log-transformed EBV load between the genotypes were performed using the Mann–Whitney test and one way Kruskal–Wallis statistic. When the *p*-value in the Kruskal–Wallis test was statistically significant, pair-wise comparisons were assessed by Dunn's test. Multivariable logistic regression analyses were performed in R, controlling for age and sex as variables influencing the risk of eBL etiology. The statistical significance of associations was assessed using odds ratios (OR) with 95% confidence intervals (CI). A *p* ≤ 0.05 was considered significant.

### Ethical approval

This research was approved by the Scientific and Ethical Review Unit (SERU) at the Kenya Medical Research Institute (KEMRI), and the Institutional Review Board at the University of Massachusetts Medical School (UMMS), Worcester, USA. All experiments were performed in accordance with relevant guidelines and regulations. Study participants were informed about the study and since they were all below 18 years, the parent and/or legal guardian provided written informed consent, before enrollment. In addition, children aged 13 years and above provided assent as per the requirements of the local IRB.

## Supplementary information

Supplementary Information 1.

Supplementary Information 2.

## Data Availability

The datasets evaluated in this study are available in supplementary data online.
