# Supplementary material for: Association of killer cell immunoglobulin-like receptors with endemic Burkitt lymphoma in Kenyan children
Source: Sci Rep. 2021 May 31;11:11343. doi: 10.1038/s41598-021-90596-7 (PMC8166913; doi:10.1038/s41598-021-90596-7)
Supplement: Supplementary file 2 — Supplementary Information 2. [file 41598_2021_90596_MOESM2_ESM.docx]

**Association of Killer Cell Immunoglobulin-like Receptors with endemic Burkitt Lymphoma in Kenyan Children.**

Beatrice M. Muriuki^1,2^, Catherine S. Forconi^3^, Peter O. Oluoch^2,3^, Jeffrey A. Bailey^4^, Anita Ghansah^5^, Ann M. Moormann^3^, and John M. Ong'echa^2*^

**^1^**West African Center for Cell Biology of Infectious Pathogens, College of Basic and Applied Sciences, University of Ghana, Accra, Ghana.

**^2^**Center for Global Health Research, Kenya Medical Research Institute, Kisumu, Kenya. **^3^**Division of Infectious Diseases and Immunology, Department of Medicine, University of Massachusetts Medical School, Worcester, MA, USA.

**^4^**Department of Pathology and Laboratory Medicine, Warren Alpert Medical School, Brown University, Providence, RI, USA.

**^5^**Noguchi Memorial Institute for Medical Research, College of Health Sciences, University of Ghana, Legon, Accra, Ghana.

Correspondence and requests for materials should be addressed to John M. Ong'echa (email:michaelongecha@yahoo.com).

**Supplemental Data**


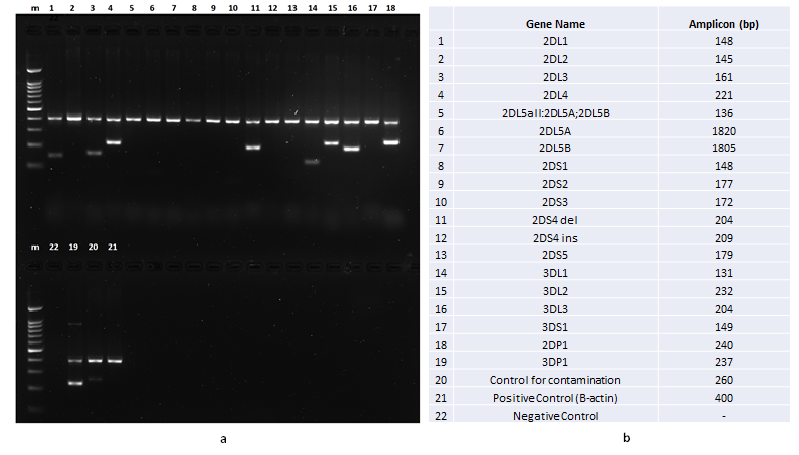


**Figure S1: a.** Agarose gel electrophoresis analysis of one sample to detect 16 KIR genes.  PCR amplified products were run on a 2% agarose gel. Lane m indicates the DNA ladder (100 bp DNA ladder, catalogue number D001, Gold Biotechnology). **b**. The gel images were interpreted according to the manufacturer's manual provided in the kit (Miltenyi, Biotec, Inc, Germany).

**Table S1. Distribution of KIRs based on centromeric and telomeric gene content**

| **Gene** | **eBL n=104 (%)** | **HC n=104 (%)** | **OR (95% CI)** | ***p* value** |
| --- | --- | --- | --- | --- |
| **Centromere** | | | | |
| cA1/ cA1 | 40 (38.5) | 46 (44.2) | 0.928 (0.477-1.808) | 0.826 |
| cA1/ cB1 | 37 (35.6) | 33 (31.7) | 0.880 (0.436-1.755) | 0.719 |
| cA1/ cB2 | 7 (6.7) | 5 (4.8) | 2.502 (0.635-10.400) | 0.191 |
| cA1/ cB3 | 13 (12.5) | 12 (11.5) | 0.961 (0.363-2.585) | 0.937 |
| cB1/cB1 | 7 (6.7) | 7 (6.7) | NA | NA |
| cB2/cB2 | 0 | 1 (0.96) | NA | NA |
| **Telomere** | | | | |
| tA1/tA1 | 97 (93.3) | 99 (95.2) | 0.594 (0.147-2.293) | 0.448 |
| tA1/tB1 | 7 (6.7) | 5 (4.8) |  |  |

**Table S1.** n represents the number of individuals; Comparisons were performed with the healthy controls as the reference group.  *p-*value less than or equal to 0.05 were considered statistically significant.  *p*≤0.05 and the OR (95% CI) were adjusted by age and sex. AA= homozygous to A. Bx= homozygous or heterozygous to B. c = centromeric region. t = telomeric region.

**Table S2. Distribution of KIR B content in the centromere (cB) and telomere (tB)**

| **Genotype** | **B**  **score** | **Centromere-Telomere** | **eBL**  **n =104 (%)** | **HC**  **n=104 (%)** | **OR (95% CI)** | ***p***  **value** |
| --- | --- | --- | --- | --- | --- | --- |
| **AA** | 0 | cAcA-tAtA | 40 (38.5) | 46 (44.2) | 0.928 (0.477-1.808) | 0.826 |
| **Bx** | 1 | cAcA-tAtB | 0 | 0 | 1.005 (0.516-1.941) | 0.988 |
|  |  | cAcB-tAtA | 52 (50.0) | 45 (43.3) |  |  |
|  | 2 | cAcA-tBtB | 0 | 0 | 1.201 (0.392- 3.579) | 0.743 |
|  |  | cAcB-tAtB | 5 (4.8) | 5 (4.8) |  |  |
|  |  | cBcB-tAtA | 5 (4.8) | 8 (7.7) |  |  |
|  | 3 | cAcB-tBtB | 0 | 0 | NA | NA |
|  |  | cBcB-tAtB | 2 (1.9) | 0 |  |  |

**Table S2.** n represents the number of individuals; Comparisons were performed with the healthy controls as the reference group. *p-*value less than or equal to 0.05 were considered statistically significant.   *p*≤0.05 and the OR (95% CI) were adjusted by age and sex. AA= homozygous to A. Bx = homozygous or heterozygous to B. c = centromeric region. t = telomeric region.
